# Implementation of a novel patient reported experience measure (PREM) in rheumatology: a cross-sectional online survey of Australian rheumatology outpatients

**DOI:** 10.1007/s00296-025-05882-8

**Published:** 2025-05-08

**Authors:** Madeleine J. Bryant, Susan Lester, Oscar Russell, Samuel Whittle, Vidya Limaye, Susanna Proudman, Rachel J. Black, Catherine L. Hill

**Affiliations:** 1 School of Medicine, Faculty of Health Sciences, University of Adelaide, Adelaide, South Australia Australia; 2 Rheumatology Unit, The Queen Elizabeth Hospital, Central Adelaide Local Health Network, 28 Woodville Road, Woodville South, SA Australia; 3 Rheumatology Unit, Royal Adelaide Hospital, Central Adelaide Local Health Network, Adelaide, SA Australia; 4 Rheumatology Research Group, Basil Hetzel Institute for Translational Health Research, 37 Woodville Road, Woodville South, South Australia Australia

**Keywords:** Patient care, Patient outcome assessment, Patient reported outcome measures, Surveys and questionnaires, Outpatients, Rheumatology

## Abstract

To determine rheumatology patient care experience using the novel Commissioning for Quality in Rheumatoid Arthritis-PREM-Australian version (CQRA-PREM-AU). Patients attending Central Adelaide Local Health Network rheumatology outpatient clinics were invited to complete a cross-sectional web-based survey, comprising CQRA-PREM-AU (22 items, 8 domains, scored 1–5), demographics, Patient Global Assessment (PGA) and Patient Reported Disease Activity (PRDA) Visual Analogue Scales (VAS) (scored 0–100), Single Item Literacy Screener (SILS). Analysed were multivariable linear regression analysis of CQRA-PREM-AU overall score with patient-related covariates (diagnosis, age, sex, SILS, PGA, PRDA), and univariate regression analysis with clinic-related covariates (site, nurse contact, type/number of consultations). Response rate was 1408/4591(31%). 214 incomplete records were excluded; 1194 records were analysed. Respondent characteristics: 68% female, median age 64 years (IQR 54, 73), 29% rural, 7% non-English language at home. PGA median score 50 (IQR 27, 63) and PRDA median score 50 (IQR 26, 69). CQRA-PREM-AU Domain 1 (regarding patient Needs and Preferences) had the best overall mean score (4.1, SD 0.86). Scored poorly were Domain 3 (Information about care, 3.5, SD 0.89), Domain 4 (Daily living, 3.5, SD 1.06) and Domain 5 (Emotional care, 3.6, SD 1.04). Worse PGA, and diagnoses of systemic lupus erythematosus, “other”, or “unknown” were all associated with significantly lower CQRA-PREM-AU score (p < 0.01). Older age, and rheumatology nurse contact were associated with better experience score (p < 0.01). Use of CQRA-PREM-AU highlights the association between contact with a rheumatology nurse and better care experience, and identifies other targets for improving healthcare delivery.

**Supplementary Information:**

The online version contains supplementary material available at 10.1007/s00296-025-05882-8.

## Introduction

Partnering with patients is a pivotal component of delivering high-quality healthcare, with Australian quality standards emphasising the importance of involving patients in the measurement and evaluation of care [1, 2]. Gathering experience-related data is essential to understand whether services are adequately equipped to address the concerns of the patients they serve. Patient Reported Experience Measures (PREMs) are surveys which capture information on the experience of receiving healthcare, specifically the process, content and impact of care, which can be described in functional and relational terms [3–8]. Patient Reported Outcome Measures (PROMs), in contrast, collect data pertaining to symptoms, disease activity and functional status, among others. Use of PREMs is frequently positioned alongside PROMS, and the combination of both data types can potentially give comprehensive insights into how healthcare is delivered and can be improved. Despite this, routine implementation of PROM data collection has thus far been more widely and systematically demonstrated than that of PREMs, across multiple surgical and medical healthcare specialties, including rheumatology (5). Increasingly, examining the patient experience is considered a fundamental component of care quality, and one that is intrinsically linked to both clinical effectiveness and patient safety [4]; use of experience-related data to improve service delivery can therefore be argued as essential both from individual stakeholder and utilitarian standpoints.

Within the Australian rheumatology landscape, there are few, if any, published data on PREM use. A rheumatology-specific PREM, the Commissioning for Quality in Rheumatoid Arthritis (CQRA)-Rheumatoid Arthritis (RA)-PREM-Australian version (CQRA-PREM-AU), is validated for use with Australian patients, building on prior work to develop and validate this instrument internationally [9–13]. Comprising 22 items (statements), CQRA-PREM-AU explores aspects of care experience including patient needs and preferences (Domain 1), coordination of care and communication (Domain 2), information, education and self-care (Domain 3), daily living (Domain 4), emotional support (Domain 5), involvement of family and friends (Domain 6), access to care (Domain 7), and overall experience (Domain 8) [9, 10]. The CQRA-PREM-AU has not previously been implemented within an actively operational health service to gather experience-related data, though its routine use has significant potential to highlight the extent to which services are meeting benchmarks for care, including the Australian Rheumatoid Arthritis Clinical Care Standard and the Australian National Safety and Quality in Healthcare Standards [14, 15]. Implementation examples of the closely related CQRA-RA-PREM instrument include the National Clinical Audit for Rheumatoid and Early Inflammatory Arthritis in the United Kingdom, and deployment in rheumatology clinical registries within the Netherlands [11, 16].

Contextually this study was conducted within rheumatology units of a health network comprising two large tertiary teaching hospitals (Hospitals 1 and 2), and a rural outreach clinic (ROC), the latter servicing a district of around 17,000 residents located 230 kms from the capital city. Residents in this district report higher-than-national average prevalence of arthritis (14.1%) and other long term health conditions (8.6%) [17]. Rheumatology outpatient care in the surveyed clinics is provided by specialist rheumatologists, supervised rheumatology trainees and specialty rheumatology nurses. Consultation types include face-to-face and telehealth, type being determined by clinical need and clinician discretion. Clinical care is supported by use of an electronic medical record (EMR) and integrated, routine use of PROMs to assess symptom burden, functional status and disease activity reported by patients attending clinic visits.

The aims of this study were to:

- explore the current care experience of patients attending outpatient rheumatology care in a tertiary health network, and
- demonstrate the feasibility of practical implementation of CQRA-PREM-AU in clinical practice.

## Patients and methods

### Study design and recruitment

Methods are reported in line with published guidance on reporting survey studies [18].

**Fig. 1:**
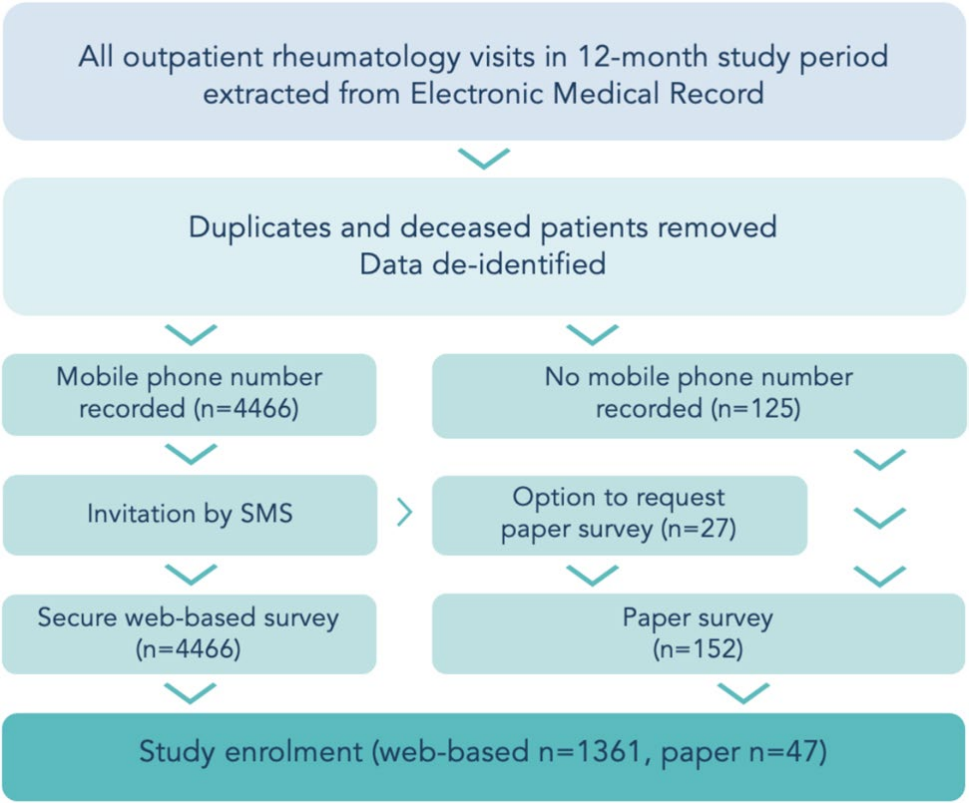
Study design

All patients attending rheumatology outpatient care at included sites between October 1 2022 and September 30 2023 were eligible to participate. Eligibility criteria were adult patients attending one or more outpatient clinic appointment. A cross-sectional single timepoint capture of all attendances in this timeframe was extracted from the EMR (Fig. 1). Duplicate attendances and deceased individuals were removed. Mobile phone numbers were extracted without other identifying details. For attendances where no mobile phone number was recorded, the EMR was hand searched for postal addresses.

Systematic sampling of all participants meeting the inclusion criteria was used. All eligible participants were invited to participate in a web-based survey via short message service (SMS), or mailed hard copy paper survey if no recorded mobile number was available, with a pre-paid return envelope. The invitation SMS was distributed using MessageMedia encrypted patient messaging service. Data collection occurred in December 2023 via the REDCap (Research Electronic Data Capture) platform. The survey link remained open for four weeks, with a reminder SMS sent at two weeks. Participation in the study was voluntary. Check-box acknowledgement of the patient information and consent form was a requirement to commence the survey. Survey responses in REDCap were anonymous and participant age was collected in decade ranges to maintain confidentiality.

### Survey content

Respondents completed 40 items: CQRA-PREM-AU (a 22-item experience measure validated for use with Australian rheumatology outpatients, with established content and face validity for this context) [10], demographic items, Patient Global Assessment (PGA) and Patient Reported Disease Activity (PRDA) Visual Analogue Scales (VAS), both scored 0–100, and Single Item Literacy Screener (SILS) scored 1–5 [19] (Supplementary Information, Document 1). CQRA-PREM-AU response options range from “strongly disagree” to “strongly agree”, scoring 1–5 per item; higher overall average score indicates a better overall care experience of the respondent [10]. For PGA VAS, higher scores represent worse overall global health assessment; for PRDA, higher scores represent more active disease [20, 21]. For SILS responses, scores > 2 identify respondents who may require health literacy support, higher scores are therefore suggestive of lower health literacy [19]. Completion time for CQRA-PREM-AU is reported at under 5 min [10].

### Analysis

Analyses included descriptive statistics, and for the CQRA-PREM-AU overall average score outcome, multivariable linear regression analysis with patient-related covariates (diagnosis, age, sex, residential location [metropolitan, rural], SILS, PGA, PRDA) and univariate analysis of clinic-related covariates (site, nurse contact, number of face-to-face clinic visits, and number of telehealth visits).

Cronbach’s alpha assessed internal consistency of CQRA-PREM-AU items in this cohort. Evidence for structural validity of a scale or subscale is a prerequisite for interpretation of internal consistency analysis (a measure of the internal structure of an instrument). Cronbach’s α ≥ 0.70 was considered indicative of sound reliability for subscale items, as well as overall scores, of the CQRA-PREM-AU.

## Results

### Respondent characteristics

The survey was distributed to 4591 eligible participants (n = 4466 by text message, n = 125 by mail). Response rate was 1408/4591 (31%); of these, 97.7% were completed online. Records were defined as complete if participants provided responses to a minimum of 2 items per Domains 1–3, and 1 item per Domains 4–8 (Appendix 1). 214 incomplete records were excluded. In total, 1194 records were included for analysis: 98% had complete CQRA-PREM-AU data, 2% had missing responses to 1–3 items.

The majority of respondents were female (n = 745, 68%), median age was 64 years (IQR 54, 73), and 72 (7%) reported speaking a non-English language at home (Table 1). Site representation was Hospital 1: n = 781 (65%), Hospital 2: n = 382 (32%), and Rural Outreach Clinic: n = 31 (3%). 315 (29%) of respondents were rurally located, comprising 31 rural respondents attending care rurally at ROC, and 285 rural respondents attending care at metropolitan sites. A broad range of reported rheumatological diagnoses were represented, reflecting the usual diagnoses seen in the clinics (Table 1).

**Table 1:** Summary demographic data of survey respondents

Characteristic	n, (%)
Sex, female: n (%)	745/1091 (68%)
Age range, years: n (%)	1105
18–40	89 (8%)
41–60	361 (32%)
61–80	575 (52%)
> / = 81	80 (7%)
Main language spoken at home: n (%)	1095
English	1023 (86%)
Other	72 (6%)
Not reported	99 (8%)
Aboriginal and/or Torres strait islander origin: n (%)	28/1104 (2.5%)
Reside in rural location^*1*^:	315/1101 (29%)
SILS: How often do you require help with pharmacy instructions?	1102
Never	786 (71%)
Rarely	153 (14%)
Sometimes	110 (10%)
Often	27 (2%)
Always	26 (2%)
Diagnosis	
Number of diagnoses	
1	883 (74%)
2	211 (18%)
> = 3	98 (8%)
Not reported	2 (0.2%)
Diagnosis breakdown	
Rheumatoid arthritis	541 (45%)
Psoriatic arthritis	118 (10%)
Ankylosing spondylitis	63 (5%)
Systemic lupus erythematosus	66 (6%)
Scleroderma	69 (6%)
Sjogren’s syndrome	60 (5%)
Enteropathic arthritis	1 (0.1%)
Vasculitis (including giant cell arteritis)	67 (6%)
Polymyalgia rheumatica	62 (5%)
Fibromyalgia	118 (10%)
Gout	64 (5%)
Osteoarthritis	157 (13%)
Don’t know	70 (6%)
Other	175 (15%)
Disease activity (PRDA) in past week (VAS): median (IQR)	50 (26, 69) n = 1079
PGA (VAS): median (IQR)	50 (27, 63) n = 1077
Treatment interactions	
Clinic attendance site: n (%)	1194
Hospital 1	781 (65%)
Hospital 2	382 (32%)
Rural outreach clinic	31 (3%)
Rheumatology nurse contact: n (%)	192/1098 (17%)
Face-to-face visits in the last 12 month: median (IQR):	2 (1, 3)
Telehealth appointments in the last 12 months: median (IQR)):	0 (0, 1)
Any telehealth in last 12 months: n (%)	450/1143 (39%)

^1^Includes rural respondents attending care at ROC and metropolitan sites

*SILS* single item literacy screener, *PRDS* patient reported disease activity, *PGA* patient global assessment, *VAS* visual analogue scale, *IQR* Interquartile range, *ROC* Rural Outreach Clinic

Patient Global Assessment median score was 50 (IQR 27, 63), and Patient Reported Disease Activity median score was also 50 (IQR 26, 69).

### Reported experiences of care

Results pertaining to reported experience of care were analysed by overall CQRA-PREM-AU score by Domain, and by individual item.

Domain 1 (regarding patient Needs and Preferences) had the best overall mean score (4.1, SD 0.86). Domain 3 (Information about care), Domain 4 (Daily living) and Domain 5 (Emotional support) all scored poorly (Domain 3 mean score 3.5, SD 0.89; Domain 4 mean score 3.5, SD 1.06; Domain 5 mean score 3.6, SD 1.04) (Table 2).

**Table 2:** Reported care experience (CQRA-PREM-AU score) by Domain, ranked

Domain	Mean score (SD)
Overall CQRA-PREM-AU score	3.77 (0.84)
Domains	
Domain 1: your needs and preferences	4.14 (0.86)
Domain 6: family and friends	4.00 (0.92)
Domain 8: overall experience of care	3.99 (1.06)
Domain 7: access to care	3.94 (1.06)
Domain 2: coordination of care and communication	3.71 (0.99)
Domain 5: emotional support	3.55 (1.04)
Domain 4: daily living	3.47 (1.06)
Domain 3: information, education and self-care	3.45 (0.89)

Items regarding respectful treatment (89% agreement), provision of information (85% agreement), and patient involvement in decision making (82% agreement) were ranked with highest agreement in this cohort (Table 3). Items addressing how to access care during a disease flare (42% agreement), referral to patient organisations or support groups (34% agreement), and referral to self-management programs (20% agreement) were ranked with the lowest agreement.

**Table 3:** Reported care experience by agreement per item, ranked

Item	Statement	Agreement (frequency “Strongly Agree” + “Agree”/Total), n	%
1a	Whenever I attended a clinic, I felt that I was treated respectfully as an individual	1063/1193	89%
1d	I was given information in a way that I could understand (eg. explained clearly or written down, in the right language for me)	1007/1190	85%
1b	I was involved as much as I wanted to be in decisions about treatment and care (eg. my medications, tests and investigations)	974/1192	82%
3a	I feel that I was given information at the time I needed it	928/1194	78%
1e	I was given enough information to help me make decisions about my treatment	927/1190	78%
3b	I feel that I have a good understanding of the treatments I am on or being offered (eg. medications, physical therapy)	917/1191	77%
8	Overall in the past year, I had had a good experience of care for my condition	924/1194	77%
6	I feel able to take a family member or support person to outpatient appointments if I want to	892/1194	75%
7a	At appointments, I feel that I have enough time with the healthcare professional to cover everything I want to discuss	909/1194	76%
2d	I feel that the people I see at the clinic are fully up to date with my current health situation	869/1191	73%
1c	My personal circumstances and preferences (eg. work or study, finances, family and carer duties, social life) were taken into account when planning and deciding on my treatment and care	833/1192	70%
4a	I feel that my condition and symptoms are being controlled enough to let me get on with my daily life and usual activities	763/1193	64%
2a	I was made aware that there is a team of health professionals looking after me, (eg. specialist doctor, GP, and may also include specialist nurses, physiotherapists, podiatrists [foot experts], occupational therapists [to assess mobility, functioning at home, and remaining active])	790/1191	63%
5a	I feel able to approach a member of my health team to discuss any worries about my condition and my treatment or their effect on my life	707/1194	59%
2c	There is a member of my health team who can help me to see other healthcare professionals in the team when needed *(*eg. referrals to other medical or surgical specialists, or physiotherapists, podiatrists, occupational therapists)	679/1190	57%
2b	When I needed help I was able to access different members of my health team	641/1194	54%
5b	I feel able to discuss personal or intimate issues about relationships with my health team if I want to	605/1192	51%
4b	If I have had a “flare” (when my symptoms get much worse), I have been able to get help quickly	506/1193	42%
3c	I have been told about patient organisations or patient support groups that can help me	401/1190	34%
3d	I have been offered an opportunity to attend a self-management or education program about my condition	241/1190	20%

### Patient factors affecting experience scores

**Fig. 2:**
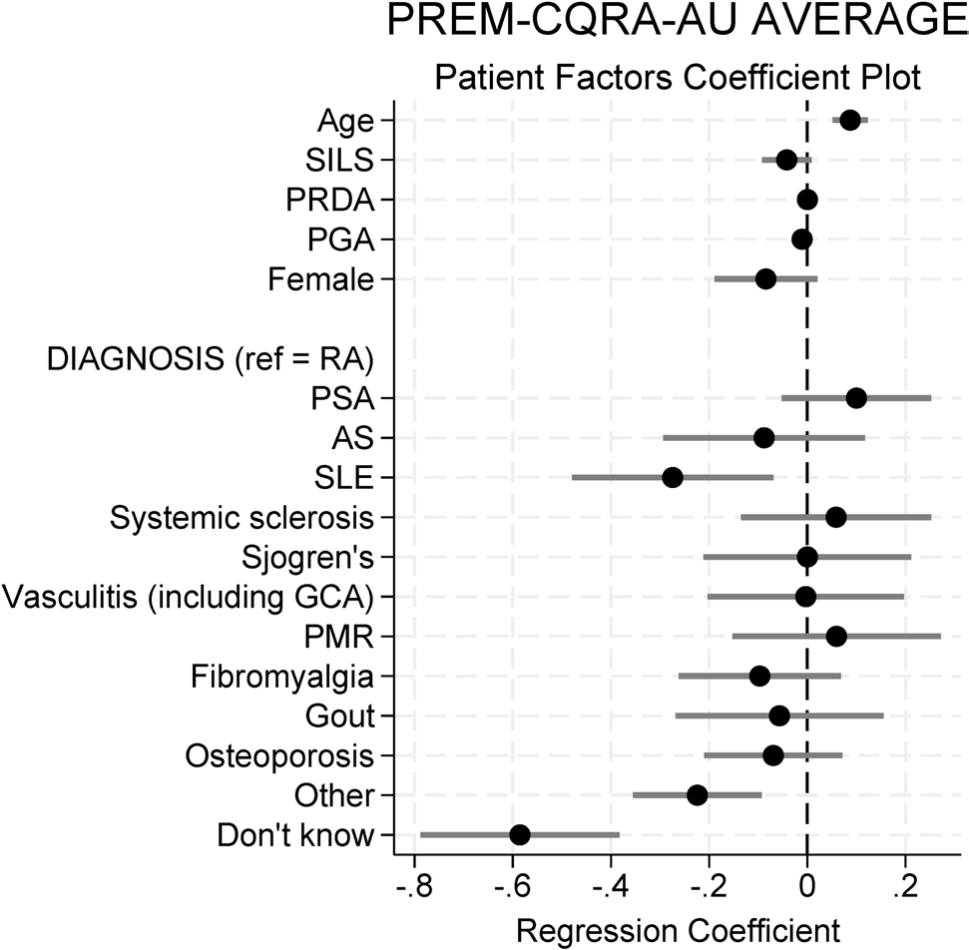
Coefficient plot for patient-related covariates. *SILS* single item literacy screener, *PRDA* patient reported disease activity, *PGA* patient global assessment, *RA* rheumatoid arthritis, *PSA* psoriatic arthritis, *SLE* systemic lupus erythematosus, *GCA* giant cell arteritis, *PMR* polymyalgia rheumatica

Patient-related covariates included in the linear regression analysis models were age range, sex, residential location, health literacy (SILS), patient-reported disease activity (PRDA), global health assessment (PGA) and diagnosis. Older age was associated with higher overall CQRA-PREM-AU score (coeff 0.09, p < 0.01) (Table 4). Higher (interpreted as poorer) score on PGA was associated with worse overall CQRA-PREM-AU score (-0.01, p < 0.01). Diagnoses of systemic lupus erythematosus (SLE) (-0.27), “Other” (-0.22) and “Don’t know” (-0.59) were all associated with worse overall CQRA-PREM-AU score (all p < 0.01) (Fig. 2). There was no significant difference in overall CQRA-PREM-AU score for sex, residential location, health literacy (SILS) or disease activity (PRDA).

**Table 4:** Linear regression coefficients for CQRA-PREM-AU overall score, by patient-related and clinic-related covariate

Overall CQRA-PREM-AU score	Coef (95% CI)	p-value	Sig
Patient-related covariates			
Age range	0.09 (0.05, 0.12)	< 0.01	***
SILS	−0.04 (−0.09, 0.01)	0.11	
Disease activity (PRDA)	0 (−0.002, 0.003)	0.80	
PGA	−0.01 (−0.013, −0.008)	< 0.01	***
Sex, female	−0.08 (−0.19, 0.02)	0.12	
Rural location^1^	0.02 (−0.09, 0.13)	0.67	
Diagnosis (reference = rheumatoid arthritis)			
Psoriatic arthritis	0.1 (−0.05, 0.25)	0.20	
Ankylosing spondylitis	−0.09 (−0.30, 0.12)	0.40	
Systemic lupus erythematosus	−0.27 (−0.48, −0.07)	< 0.01	***
Scleroderma	0.06 (−0.14, 0.25)	0.55	
Sjogren’s syndrome	0 (−0.211, 0.212)	1.0	
Vasculitis	0 (−0.02, 0.20)	0.98	
Polymyalgia rheumatica	0.06 (−0.15, 0.27)	0.58	
Fibromyalgia	−0.10 (−0.26, 0.07)	0.25	
Gout	−0.06 (−0.27, 0.16)	0.60	
Osteoarthritis	−0.07 (−0.21, 0.07)	0.33	
Other	−0.22 (−0.36, −0.09)	.001	***
Don’t know	−0.59 (−0.79, −0.38)	< 0.01	***
Clinic-related covariate: site			
Hospital 1	0		
Hospital 2	−0.01 (−0.11, 0.09)	0.87	
Rural Outreach Clinic	−0.33 (−0.63, −0.03)	0.03	**
Clinic-related covariate: rheumatology nurse contact			
Nurse contact: no	0		
Nurse contact: yes	0.28 (0.15, 0.41)	< 0.01	***
Clinical consultations by number and type			
Higher number of face-to-face	0.06 (0.03, 0.10)	< 0.01	***
Higher number of telehealth	0.02 (−0.02, 0.07)	0.298	

Note 1: Includes rural respondents attending care at rural and metropolitan sites

***p < *0.01*, **p < *0.05*

*SILS* single item literacy screener, *PRDA* patient reported disease activity, *PGA* patient global assessment

### Clinic factors affecting experience scores

Attendance at the ROC, compared with other sites, was associated with worse overall CQRA-PREM-AU score (coeff -0.33, p < 0.05). Despite this finding, correlation of overall CQRA-PREM-AU score with covariate rural residential status was not significant, irrespective of adjustment (rural respondents attending care at ROC [n = 31, 10%, p = 0.33]) or unadjusted (rural respondents attending care at a metropolitan site, [n = 284, 90%, p = 0.67]). Rural residental status was thus not predictive of overall CQRA-PREM-AU scores.

Contact with a specialty Rheumatology nurse was associated with higher overall CQRA-PREM-AU score (coeff 0.28, p < 0.01). A higher number of face-to-face clinic visits in the preceding 12 months was associated with higher overall CQRA-PREM-AU score (coeff 0.06, p < 0.01), while higher number of telehealth appointments had no significant effect on overall CQRA-PREM-AU score (coeff 0.02, p = 0.3).

### Clinic cancellations

307 (26%) respondents indicated they had had clinic appointments cancelled unexpectedly. Wait time for a rescheduled appointment was more than 1 month for 44%, and more than 3 months for 23%.

### Instrument reliability

The CQRA-PREM-AU demonstrated internal consistency in this dataset with Cronbach’s alpha scores > 0.7 for all domains (Supplementary Information, Document 2) and 0.97 for the overall average score. Additionally, Cronbach’s alpha scores were > 0.7 for all domains reported by respondents indicating English as a second language (n = 72).

## Discussion

This study presents a current picture of patient care in selected Australian public rheumatology clinics. The novel CQRA-PREM-AU experience measure, validated for appraisal of self-reported care experience in Australian rheumatology patients, was deployed in an operational health service with a mixed patient cohort for the first time [10]. There is a strong case for routine PREM capture in rheumatology, this cohort of patients being likely to require frequent and long-term specialty input, and bear the impact of rheumatological diagnoses on quality of life. Several instruments have been developed for this purpose internationally [9, 11, 12, 22–25], and previously described in this journal [22]. CQRA-RA-PREM and its derivatives remain the most widely reported in published literature. This study builds on existing examples of how PREM capture can be practically implemented and used to collect meaningful data to improve personalised care, service development, and engagement with patients.

Reassuringly, patients attending this service demonstrate agreement with statements about receiving respectful care (89% agreement), receiving information and resources (85% agreement), and being involved in decision making (82% agreement), qualities in accord with the Australian Charter of Healthcare Rights and a recent report on priorities of Australian rheumatology patients [26, 27]. Despite this, ranking by item did not reflect overall ranking of composite Domains, most significantly because of low endorsement of having been referred to patient support groups and self-management programs (comprising items in Domain 3, 34% and 20% agreement respectively). Formal referral pathways to self-management programs for rheumatology patients are lacking within the studied region, though attendance at a self-management patient program launched in this network within the study period has been correlated with improved self-efficacy and daily function (pre-publication data). These findings confirm a gap in service and a high priority for expansion, and can potentially be used to support funding proposals for models of care to expand self-management and educational streams to have measurable impact on quality of care experience and patient outcomes. Participation in this study was a significant source of referral for the self-management program, suggesting that campaigns to raise visibility and awareness of such resources would be another readily modifiable way to improve care experience in this region.

Of concern, reported experience of Domain 4 (regarding Daily living) was low in this cohort (mean score 3.5), predominantly due to patients reporting low agreement (42%) with knowing how to get help during a flare. The low ranking of this Domain, which also encompasses an item concerning disease control, was consistent with higher-than-expected PRDA VAS scores (median 50, IQR 26–69). Prior qualitative work with rheumatology patients has demonstrated the high importance placed by patients on knowing how to access inter-visit and flare care between clinic visits [27], a finding underscored by these data. A further key outcome from this study is the correlation between contact with a specialty rheumatology nurse and better overall care experience. Services provided by specialty rheumatology nurses include clinic assessments, education, support around medication access, and offering a key point of contact for inter-visit and flare care, among many others [28–30]. It is therefore not surprising that nurse contact was a predictor of better care experience in this cohort. The obvious challenge then is to consider how this service can be extended to all patients attending care within networks, as specialty nurses are not currently available for all clinics across the service in question, nor are they able to have contact with every patient within each of these clinics (5/8 weekly clinics at Hospital 1, 5/7 weekly clinics at Hospital 2, no clinics at Rural Outreach Clinic). Enhanced access to specialty nursing, and improved flare care conceivably are readily modifiable clinic-related factors identified by this study. Specific interventions targeted to improve mutual engagement of clinicians and patients with respect to these issues will be audited in future quality improvement cycles.

Notable associations between patient-related factors and poor overall care experience in this cohort were diagnosis of SLE, diagnosis “Other” and diagnosis “Unknown”, in addition to higher PGA score. These diagnostic groups are likely heterogeneous in terms of disease phenotype and severity, and may represent those with diagnostic uncertainty or prolonged time to definitive diagnosis. These groups may benefit from targeted interventions such as offering patient education, ensuring diagnoses are communicated clearly to patients, and follow up exploration of their experiences.

Results demonstrating that attendance at ROC was associated with poorer overall care experience require specific discussion. Several clinic-specific factors are relevant to interpretation of this finding. Of note, this clinic was established only at the beginning of 2022, less than 12 months prior to the survey period. Secondly, it is staffed largely by rotating clinicians, with variable arrangements for inter-visit care of patients, owing to considerable distance from the metropolitan centre. Thirdly, no specialty nursing support is part of the current model of care. All these factors pose a challenge to continuity of care. Notably, while attendance at ROC in this survey period correlated with poorer overall experience, self-reported rural location of patients was not a significant predictor, suggesting that it is clinic-related factors of the specific ROC which need to be modified in order to improve this outcome in the future.

Strengths of this study included its reach and response rate. Invitations to participate sent to all patients attending care within the service within a 12-month period, and a mixed online and paper model for data collection was used to maximise inclusion and limit potential response bias. A response rate of 31% is considered a strength: by comparison, response rates to equivalent experience measures, such as the Hospital Consumer Assessment of Healthcare Providers and Systems (HCAHPS) in the United States, are reported between 26–33% [31]. This study is also noteworthy in that it reports the implementation of the novel CQRA-PREM-AU following adaptation and validation for use with Australian patients. The overall (average) CQRA-PREM-AU score has not previously been reported, and its use in this study illustrates how it can be used to interpret experience scores both by Domain, and also to describe differing overall experience by patient- and clinic-related factors. The high Cronbach α values for CQRA-PREM-AU domains and overall score indicate reliability of the instrument in this cohort, which included patients with multi-system inflammatory disorders such as SLE and systemic sclerosis, diagnoses not represented in prior validation work [10]. These findings suggest that no further revalidation with Australian mixed rheumatology cohorts is required. Additionally, while the number of respondents reporting English as a second language at home were relatively small in this sample (n = 72), again the high calculated Cronbach α values for this group confirm reliability of the instrument when used with non-English speaking cohorts, as a novel finding. Deployment of the survey was conducted using existing clinical software (EMR, MessageMedia and RedCap) with no additional consumable costs: this is a clear benefit given that funding limitations and resource allocation are frequently cited barriers to implementation of PREM use in non-rheumatology contexts [32–35]. The rate-limiting step to dissemination of findings was data analysis, a process which could be readily streamlined in future implementation cycles for greater efficiency, supporting the assertion that routine use of CQRA-PREM-AU is a feasible method of regularly assessing care experience.

### Limitations

Several limitations are acknowledged in this study. Firstly, due to the anonymised study design, demographic data for non-respondents was not collected, making comparison between cohorts and assessment for responder bias impossible. As such, there is a possibility of responder bias impacting the interpretation of results, which may have important implications if outcomes are used to effect changes in service delivery. While this phenomenon has been explored as an issue in interpretation of satisfaction surveys, published guidance is lacking on whether responder bias is likely to be a significant problem in collection of experience data [36]. This methodological approach was weighed against the preference for anonymity of respondents as a priority, to optimise response rate. Secondly, respondents were able to select multiple applicable diagnoses, rather than diagnosis by hierarchy, making sub-analyses by diagnosis cohort challenging. Data on disease duration were not collected; this may have relevance in terms of differing care experience between newly diagnosed and longer term patients. Thirdly, there has been no prior guidance on the implication of missing data for the interpretation of CQRA-PREM-AU. In this study, pre-determined criteria were set for inclusion of responses to ensure all instrument Domains were answered (Appendix 1), on the basis that high inter-relatedness of items (Cronbach’s alpha values) affords capacity to accommodate some missing responses without compromising interpretation of the overall score. The minimum included item response rate in this dataset was 17/20 items. On this basis, a minimum response rate of 16/20 items (80% completion) is proposed as acceptable in future instrument use, noting also that this issue could be circumnavigated using an online format requiring all items to be answered in order to progress through the survey. Lastly, the lack of longitudinal data for comparison is acknowledged. Future studies will focus on repeating network-wide collection of patient experience data in South Australia, enabling comparison of findings before and after service improvements.

## Conclusion

In summary, this study identifies targets for improving patient experience in specific care domains and for certain patient groups, using CQRA-PREM-AU in a mixed rheumatology cohort. Key findings are the correlation between contact with a specialty rheumatology nurse and better reported care experience, and that respectful, patient-centred care is reported by this sample. Higher (worse) PGA score, uncertain or unknown diagnosis, and SLE were found to be correlates of poorer care experience, and respondents indicated poor agreement with ready access to flare care, referral to support groups and self-management resources. Overall this study highlights key targets for focused efforts to improve service delivery and improve the care experience of Australian rheumatology patients.

## Supplementary Information

Below is the link to the electronic supplementary material.

Supplementary file1 (DOCX 53 KB)

Supplementary file1 (DOCX 574 KB)

## Data Availability

The data underlying this article will be shared on reasonable request to the corresponding author.

## Declarations

### Human and animal right

Where applicable, the authors confirm that all human and animal studies have been approved by the appropriate ethics committee and have therefore been performed in accordance with the ethical standards laid down in the 1964 Declaration of Helsinki and its later amendments.

### Patient participation

A patient representative reviewed and approved the Patient Information and Consent Form and study protocol.
