# Supplementary material for: Implementation of a novel patient reported experience measure (PREM) in rheumatology: a cross-sectional online survey of Australian rheumatology outpatients
Source: Rheumatol Int. 2025 May 8;45(6):138. doi: 10.1007/s00296-025-05882-8 (PMC12062103; doi:10.1007/s00296-025-05882-8)
Supplement: Supplementary file 1 — Supplementary file1 (DOCX 53 KB) [file 296_2025_5882_MOESM1_ESM.docx]

**JOURNAL**

Rheumatology International

**TITLE**

IMPLEMENTATION OF A NOVEL PATIENT REPORTED EXPERIENCE MEASURE (PREM) IN RHEUMATOLOGY: A CROSS-SECTIONAL ONLINE SURVEY OF AUSTRALIAN RHEUMATOLOGY OUTPATIENTS

**AUTHORS**

Madeleine J Bryant^1,2,3^, Susan Lester^1,2,4^, Oscar Russell^1,2,4^, Samuel Whittle^1,2,4^, Vidya Limaye^1,3^, Susanna Proudman^1,3^, *Rachel J Black^1,2,3,4^, *Catherine L Hill^1,2,3,4^

**AFFILIATIONS**

(1) School of Medicine, Faculty of Health Sciences, University of Adelaide, Adelaide, South Australia, Australia.

(2) Rheumatology Unit, The Queen Elizabeth Hospital, Central Adelaide Local Health Network, Adelaide, South Australia, Australia.

(3) Rheumatology Unit, Royal Adelaide Hospital, Central Adelaide Local Health Network, Woodville South, South Australia, Australia.

(4) Rheumatology Research Group, Basil Hetzel Institute for Translational Health Research, The Queen Elizabeth Hospital, Woodville South, South Australia, Australia.

**CORRESPONDING AUTHOR**

Madeleine J Bryant

[Madeleine.bryant@sa.gov.au](mailto:Madeleine.bryant@sa.gov.au)

Rheumatology Unit, The Queen Elizabeth Hospital, 28 Woodville Road, Woodville South, South Australia 5011

ORCID 0000-0002-7946-8620

**Supplementary Document 1: Survey protocol**

| INTRODUCTION | | |
| --- | --- | --- |
|  | Thank you for participating in this short survey to help us understand what works well in the rheumatology clinic, and which areas need improvement.  We would like to know about your experiences when you come to clinic.  We estimate that the survey will take no longer than 10 minutes to complete.  Please complete the survey only once.    This is an anonymous survey and your participation is optional. It will not impact on your rheumatology care in any negative way.  The survey has been registered via the CALHN quality improvement office.  The project coordinator is Dr Madeleine Bryant.  If you have any questions please contact staff in the rheumatology department at the site where you attend appointments:  RAH: phone number  TQEH or Port Pirie: phone number  CALHN Research Services: phone number  Thank you for your time. |  |
| CONSENT | | |
|  | By continuing with the survey, you acknowledge that you give consent to take part and for the information provided to be collected and included in de-identified review. I give my consent: | Yes |
|  | I would like to participate in this survey, but I would prefer to receive a paper copy in the mail. If so please enter your name and postal address:  If you are willing to continue the online format, please leave this section blank | Free Text: |
| ABOUT YOUR CARE | | |
|  | Thinking about the majority of your rheumatology appointment/s in the past year, where did you attend rheumatology clinic? | Royal Adelaide Hospital- RAH main hospital  RAH – North Terrace  CALHN East – Wakefield Street  The Queen Elizabeth Hospital  Port Pirie |
|  | Thinking about the majority of your appointment/s, what day of the week did you attend clinic? | Monday morning  Monday afternoon  Tuesday morning  Tuesday afternoon  Wednesday morning  Wednesday afternoon  Thursday morning  Thursday afternoon  Friday morning  Friday afternoon  Can’t recall |
|  | How many face-to face visits to the rheumatology clinic have you had in the past 12 months (give approximate) | 1  2  3  4  5  6  7  8 or more |
|  | How many phone or telehealth appointments have you had with the rheumatology clinic in previous 12 months (give approximate)? | I haven’t had any phone or telehealth appointments  1  2  3  4  5  6  7  8 or more  Can’t recall |
|  | Which rheumatic disease are you being treated for? (please select all that apply) | Rheumatoid Arthritis  Psoriatic Arthritis  Ankylosing Spondylitis  Systemic Lupus Erythematosus  Scleroderma  Sjogren’s syndrome  Enteropathic arthritis  Vasculitis (including Giant Cell Arteritis)  Polymyalgia rheumatica  Fibromyalgia  Gout  Osteoarthritis  Don’t know  Other |
| CQRA-PREM-AU | | |
|  | Whenever I attended a clinic, I felt that I was treated respectfully as an individual | Strongly agree  Agree  Neither agree nor disagree  Disagree  Strongly disagree |
|  | I was involved as much as I wanted to be in decisions about treatment and care (*eg. my medications, tests and investigations)* | Strongly agree  Agree  Neither agree nor disagree  Disagree  Strongly disagree |
|  | My personal circumstances and preferences (*eg. work or study, finances, family and carer duties, social life)* were taken into account when planning and deciding on my treatment and care | Strongly agree  Agree  Neither agree nor disagree  Disagree Strongly disagree |
|  | I was given information in a way that I could understand (*eg. explained clearly or written down, in the right language for me)* | Strongly agree  Agree  Neither agree nor disagree  Disagree  Strongly disagree |
|  | I was given enough information to help me make decisions about my treatment | Strongly agree  Agree  Neither agree nor disagree  Disagree  Strongly disagree |
|  | I was made aware that there is a team of health professionals looking after me, (*eg. specialist doctor, GP, and may also include specialist nurses, physiotherapists, podiatrists [foot experts], occupational therapists [to assess mobility, functioning at home, and remaining active]*) | Strongly agree  Agree  Neither agree nor disagree  Disagree  Strongly disagree |
|  | When I needed help I was able to access different members of my health team | Strongly agree  Agree  Neither agree nor disagree  Disagree  Strongly disagree |
|  | There is a member of my health team who can help me to see other healthcare professionals in the team when needed *(eg. referrals to other medical or surgical specialists, or physiotherapists, podiatrists, occupational therapists)* | Strongly agree  Agree  Neither agree nor disagree  Disagree  Strongly disagree |
|  | I feel that the people I see at the clinic are fully up to date with my current health situation | Strongly agree  Agree  Neither agree nor disagree  Disagree  Strongly disagree |
|  | I feel that I was given information at the time I needed it | Strongly agree  Agree  Neither agree nor disagree  Disagree  Strongly disagree |
|  | I feel that I have a good understanding of the treatments I am on or being offered (*eg. medications, physical therapy)* | Strongly agree  Agree  Neither agree nor disagree  Disagree  Strongly disagree |
|  | I have been told about patient organisations or patient support groups that can help me | Strongly agree  Agree  Neither agree nor disagree  Disagree  Strongly disagree |
|  | I have been offered an opportunity to attend a self-management or education program about my condition | Strongly agree  Agree  Neither agree nor disagree  Disagree  Strongly disagree |
|  | I feel that my condition and symptoms are being controlled enough to let me get on with my daily life and usual activities | Strongly agree  Agree  Neither agree nor disagree  Disagree  Strongly disagree |
|  | If I have had a “flare” (when my symptoms get much worse), I have been able to get help quickly | Strongly agree  Agree  Neither agree nor disagree  Disagree  Strongly disagree |
|  | I feel able to approach a member of my health team to discuss any worries about my condition and my treatment or their effect on my life | Strongly agree  Agree  Neither agree nor disagree  Disagree  Strongly disagree |
|  | I feel able to discuss personal or intimate issues about relationships with my health team if I want to | Strongly agree  Agree  Neither agree nor disagree  Disagree  Strongly disagree |
|  | I feel able to take a family member or support person to outpatient appointments if I want to | Strongly agree  Agree  Neither agree nor disagree  Disagree  Strongly disagree |
|  | At appointments, I feel that I have enough time with the healthcare professional to cover everything I want to discuss | Strongly agree  Agree  Neither agree nor disagree  Disagree  Strongly disagree |
|  | I have had clinic appointments cancelled unexpectedly  If yes, how long have you had to wait for a new appointment? | Yes  No  Unsure  Less than 1 month  1-3 months  More than 3 months  Unsure |
|  | I have needed extra treatment or a change of treatment between routine appointments  If yes, how long did it take for this to happen? | Yes  No  Unsure  Less than 1 month  1-3 months  More than 3 months  Unsure |
|  | Overall in the past year, I have had a good experience of care for my condition | Strongly agree  Agree  Neither agree nor disagree  Disagree  Strongly disagree |
| Your current health: | | |
|  | In the past week, how active has your rheumatology disease been? | Visual Analogue Scale  Mark a line along the scale below to indicate your response from not active to extremely active  Not active 0mm – Extremely active 10mm |
|  | Considering all the ways in which illness and health conditions may affect you at this time, please indicate below how you are doing: | Visual Analogue Scale:  Mark a line along the scale below to indicate your response from very well to very poorly  Very well 0mm - Very poorly 100mm |
| A few more questions about you: | | |
|  | Are you: | Male  Female  Non-binary  Prefer not to say |
|  | What is your age range? | 18-30  31-40  41-50  51-60  61-70  71-80  81-90  90 or older |
|  | What is the main language you speak at home? | English  Other: please specify |
|  | Language if other than English: | Free text: |
|  | How often do you need to have someone help you when you read instructions, pamphlets, or other written material from your doctor or pharmacy? | Never  Rarely  Sometimes  Often  Always |
|  | Are you of Aboriginal and/or Torres Strait Islander origin? | No  Aboriginal  Torres Strait Islander  Both Aboriginal and Torres Strait Islander |
|  | Do you live in a rural location? | Yes. I live in a rural location  No. I live in metropolitan or greater Adelaide |
|  | Have you had education or additional phone calls with the rheumatology nurse? | Yes  No |
|  | I am satisfied with my care at the rheumatology clinic | Strongly agree  Agree  Neither agree nor disagree  Disagree  Strongly disagree |
|  | Do you have anything else that you would like to tell us or suggestions about how the rheumatology clinic works well, or could be improved? | Free text |
|  | Are you interested in being part of our consumer group aimed at improving the quality of our services for rheumatology patients? If so, please leave your name and a phone number of email address you can be contacted on. This is entirely voluntary. If you are not interested, please leave this section blank. | Free text |
|  | Our allied health team runs an 8-week group program to support the physical and mental wellbeing of rheumatology patients. It is free to attend. If you are interested in finding out more, please leave your name and phone number or email address and the team will get in touch. This is entirely voluntary. If you are not interested, please leave this section blank. | Free text |

S**upplementary Document 2:**

**Table 5: Cronbach’s alpha for CQRA-PREM-AU items.**

| Domain | Description | Items | Items, n | α, all patients (n=1194) | α, non-English language spoken (n=72) |
| --- | --- | --- | --- | --- | --- |
| Domain 1 | Needs and preferences | 1a, 1b, 1c, 1d, 1e | 5 | 0.93 | 0.93 |
| Domain 2 | Co-ordination of care and communication | 2a, 2b, 2c, 2d | 4 | 0.94 | 0.93 |
| Domain 3 | Information, education and self-care | 3a, 3b, 3c, 3d | 4 | 0.85 | 0.86 |
| Domain 4 | Daily living | 4a, 4b | 2 | 0.77 | 0.73 |
| Domain 5 | Emotional support | 5a, 5b | 2 | 0.85 | 0.89 |
| Domain 6 | Family and friends | 6 | 1 | NA | NA |
| Domain 7 | Access to care | 7a | 1 | NA | NA |
| Domain 8 | Overall experience of care | 8 | 1 | NA | NA |
| Overall | CQRA-PREM-AU overall score |  | 20 | 0.97 | 0.97 |
| Key: CQRA-PREM-AU Commissioning for Quality in Rheumatoid Arthritis-Patient Reported-Experience-Measure-Australian version | | | | | |
