# Supplementary material for: Implementation of a novel patient reported experience measure (PREM) in rheumatology: a cross-sectional online survey of Australian rheumatology outpatients
Source: Rheumatol Int. 2025 May 8;45(6):138. doi: 10.1007/s00296-025-05882-8 (PMC12062103; doi:10.1007/s00296-025-05882-8)
Supplement: Supplementary file 2 — Supplementary file1 (DOCX 574 KB) [file 296_2025_5882_MOESM2_ESM.docx]

**TITLE:**

IMPLEMENTATION OF A NOVEL PATIENT REPORTED EXPERIENCE MEASURE (PREM) IN RHEUMATOLOGY: A CROSS-SECTIONAL ONLINE SURVEY OF AUSTRALIAN RHEUMATOLOGY OUTPATIENTS

**APPENDIX 1: CQRA-PREM-AU**


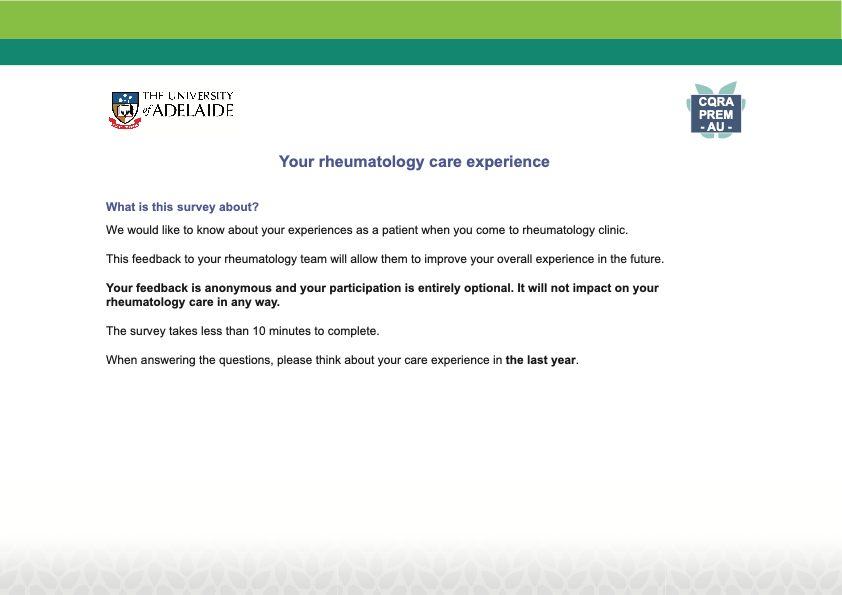


**
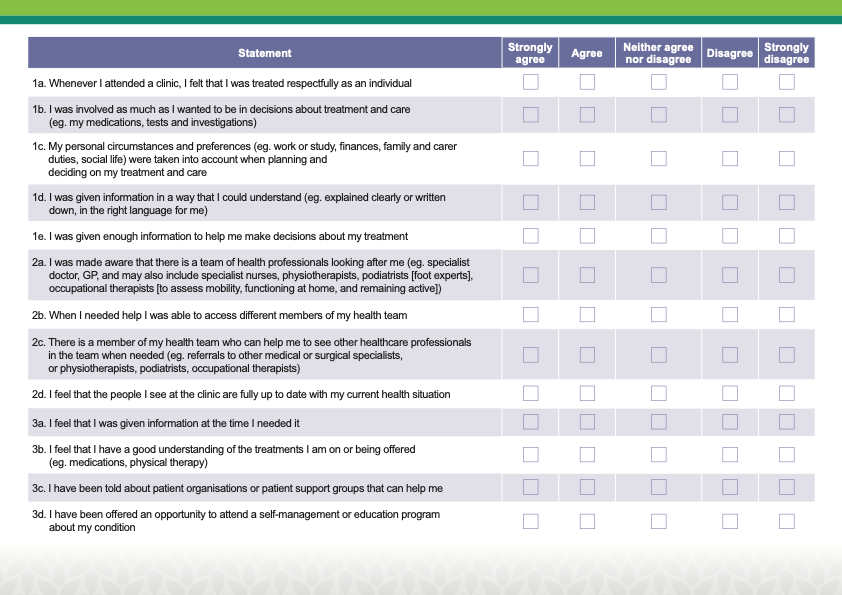

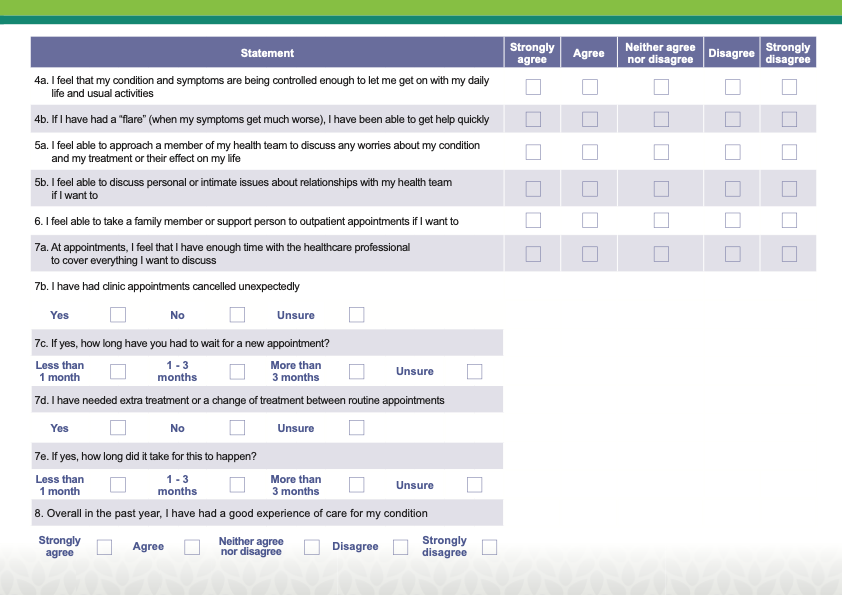

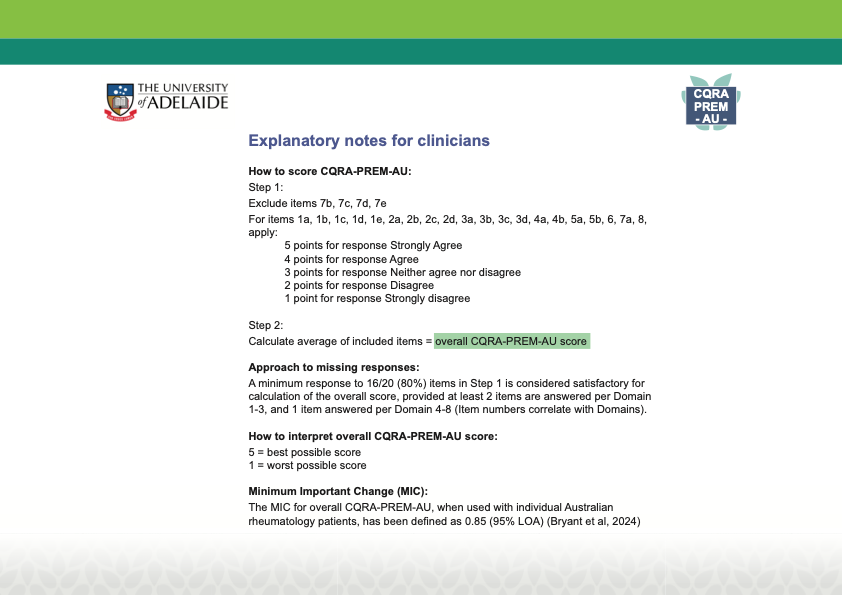
**
